# Why Kinematic Alignment Makes Little Sense in Valgus Osteoarthritis of the Knee: A Narrative Review

**DOI:** 10.3390/jcm13051302

**Published:** 2024-02-25

**Authors:** Christian Manuel Sterneder, Martin Faschingbauer, Lyubomir Haralambiev, Maximilian F. Kasparek, Friedrich Boettner

**Affiliations:** 1 Adult Reconstruction and Joint Replacement Department, Hospital for Special Surgery, 535 East 70th Street, New York, NY 10021, USA; 2 Department of Orthopedic Surgery, University of Ulm, Oberer Eselsberg 45, 89081 Ulm, Germany; 3 Center for Orthopaedics, Trauma Surgery and Rehabilitation Medicine, University Medicine Greifswald, Ferdinand-Sauerbruch-Straße, 17475 Greifswald, Germany; 4 Department of Orthopedics, Evangelisches Krankenhaus, Hans-Sachs Gasse 10-12, 1180 Vienna, Austria

**Keywords:** valgus osteoarthritis, valgus knee, mechanical alignment, kinematic alignment, total knee arthroplasty, knee osteoarthritis

## Abstract

There is a debate about the best alignment strategies in total knee arthroplasty (TKA). Mechanical alignment (MA) targets in combination with necessary soft tissue releases are the gold standard for TKA in end-stage valgus osteoarthritis. Some authors propagate kinematic alignment (KA) with the aim of restoring the patient’s native alignment and minimizing the need for soft tissue releases. Our previous studies showed that MA with standardized soft tissue release produces reproducible results, and that the preoperative phenotype does not influence the results of patients with valgus osteoarthritis. These data suggest that there is no functional advantage to preserving valgus alignment in patients with valgus osteoarthritis. Many patients with valgus osteoarthritis present with a compromised medial collateral ligament and leaving the knee in valgus could increase the risk of secondary instability. The current literature supports MA TKA with soft tissue release as the gold standard. While using more sophisticated enabling technologies like robotic surgery might allow for aiming for very slight (1–2°) valgus alignment on the femoral side, any valgus alignment outside this range should be avoided. This review paper summarizes our current knowledge on the surgical techniques of TKA in patients with valgus osteoarthritis.

## 1. Introduction

Approximately 15% of patients undergoing total knee arthroplasty (TKA) present with valgus osteoarthritis [1]. Both bone remodeling and soft tissue changes characterize valgus osteoarthritis [2]: a contracted posterolateral capsule, a contracted iliotibial band (ITB), a contracted lateral collateral ligament (LCL), a contracted posterior cruciate ligament (PCL), medial collateral ligament (MCL) laxity, osseous deficiency of the posterior lateral femoral condyle and/or posterolateral tibial plateau, external rotation of the distal femur and patellar maltracking are the anatomic features of valgus osteoarthritis [3,4,5,6].

There are three grades of severity according to the Ranawat classification depending on the degree of medial laxity [3]. Type I is characterized by minimal valgus deformity and medial soft tissue stretching [3]. Type II represents a fixed valgus deformity with more substantial deformity (>10°) and medial soft tissue stretching [3] and type III shows severe deformity of both the lateral femoral condyle and lateral tibia plateau with an incompetent medial soft tissue sleeve and pronounced contracted lateral structures [3,7].

In the early stages of valgus osteoarthritis, conservative treatment is the preferred treatment and includes exercise, weight loss, pharmacological treatments (NSAIDs) and unloader braces [8]. In addition, there appears to be a growing interest in alternative therapies, including acupuncture, PRP injections, stem cell injections and dry needling [9,10,11,12]. A recent systematic review and meta-analysis showed very low-quality evidence for a possible positive short-term effect on osteoarthritis concerning pain intensity and improving physical function [13]. Data on dry needling are controversial; while some authors reported short-term analgesic effects and additional benefits when combined with physical therapy [14], others reported no additional pain reduction for patients with osteoarthritis of the knee when dry needling was combined with an exercise program [15]. Nevertheless, when there is radiological confirmation of bone-on-bone osteoarthritis without an adequate response to non-operative treatments, surgical treatment with TKA is usually recommended [16].

### 1.1. Current Aligment Strategies for TKA

There is a debate about the best alignment strategy in TKA. Mechanical alignment (MA) is considered to be the gold standard [17,18,19]. Insall et al. popularized this alignment strategy with the goal of a neutral alignment of the lower extremity (straight leg axis) [20,21]. The femoral and tibial components are aligned perpendicular to the mechanical axis [20,22]. MA leads to an equal loading of the prosthesis and improves its long term outcomes [22,23,24]. These considerations have been confirmed by biomechanical studies [25]. In general, the advantages of MA in TKA are its simplicity and reproducibility; it is a well-known surgical technique in combination with excellent long-term results [17,26,27]. One point of criticism is that there is still a dissatisfaction rate of up to 20% [28,29,30] in patients undergoing total knee arthroplasty; however, most reports focus on varus osteoarthritis and satisfaction rates for patients undergoing total knee arthroplasty following valgus OA are rare.

Some authors blame MA for these high rates of dissatisfaction, because MA does not take the native phenotype into account [17,31,32,33]. They propagate kinematic alignment (KA), which is a femur-first, gap-balancing approach, with restoration of the orientation of the tibio-femoral joint line and therefore minimal need for soft tissue releases [31,32,34,35,36]. True measured resection is the basis for KA and inverse KA [31,37,38,39]. For this purpose, cartilage wear is taken into account: the goal is to resect as much bone and cartilage as is replaced by the components [20,40]. The position of the components tries to restore the native medial and lateral compartment forces, Q-angle and hip–knee–ankle (HKA) [20,41]. In KA, the need for ligament release is minimized [20]. In general, authors who support KA see benefits in restoring natural ligament tension and the need for fewer soft tissue releases with a possible advantage in terms of pain reduction, improved early function and mobility and faster rehabilitation [20].

A variant of KA with the goal of restoring native alignment within ± three degrees of neutral is restricted kinematic alignment (rKA) [42]. When these limits are exceeded, the angles are adjusted until they fall into the boundaries of rKA in order to avoid extreme positions of the implants [42,43,44].

The introduction of robotic instruments has enhanced the measurement and management of implant positioning and balancing goals [45]. These technologies allow for preoperative planning using imaging modalities that enable an evaluation of bone architecture in all three planes to customize implant size and positioning [46]. In addition, with the use of these technologies, soft tissue balancing can be quantitatively evaluated during surgery [46]. Using robotic devices, functional alignment (FA) is an alignment strategy that attempts to restore three-dimensional constitutional alignment with respect to the individual soft tissues of each patient [45,46]. This method combines components of gap-balancing and measured resection [47]. FA is characterized by the following (1.–6.): (1.) preoperative planning, (2.) restoring of native coronal alignment, (3.) reconstitution of dynamic sagittal alignment (boundaries of five degrees to normal values), (4.) preservation of joint line obliquity (JLO) and height, (5.) matching the anatomy with the implants [46], (6.) rather than using soft tissue releases, achieving a balanced joint through the manipulation of the implant’s position [46]. Placing implants in a way that least impairs soft tissues is the aim of functional alignment [47]. Ligament release may be necessary in cases of fixed deformities [47]. However, releases are required less often and the extent of the releases is supposed to be less in comparison to MA [47].

Inverse kinematic alignment (iKA) is a recently presented philosophy for patient-specific alignment in TKA [48]. Restoring the native tibial JLO is the goal of this tibia-first, gap-balancing approach [49]. Through bone and cartilage removal from the medial and lateral tibial condyles in accordance with the size of the components, the original tibial anatomy is restored [49]. The next steps of this technique follow the conventional approach, aiming for a balanced knee, including gap balancing in extension and flexion by adjusting femoral cuts [49]. Soft tissue releases are not carried out inside values of the HKA from 174 to 183 degrees [49].

In iKA, balancing the knee is performed by adjusting the orientation of femoral cuts, whereas in KA, balancing the knee is performed by adjusting the orientation of the tibial cut [49]. Winnock de Grave et al. found comparable results in varus, neutral and valgus knees for restricted iKA compared to adjusted MA TKA [48]. Another study reported increasing satisfaction rates at one-year follow-up for restricted iKA for all knee types [50].

### 1.2. Standards in Surgery for TKA in Valgus Osteoarthritis

The valgus knee has challenged surgeons for many years and detailed reports on the recommended surgical technique exist. **Whiteside** recommends a mechanical aligned TKA (MA TKA) for the valgus knee followed by soft tissue release to balance the soft tissues [51,52]. Distal femoral cuts are made in 5° valgus to the long axis of the femur, perpendicular to the mechanical axis of the femur and parallel to the epicondylar axis [51,52]. Anterior and posterior cuts of the femur are made perpendicular to the AP axis of the femur [52]. The tibial surface is cut perpendicular to the long axis of the tibia [51].

**Ranawat** recommends avoiding an undercorrection of the existing deformity by utilizing a 90° proximal tibial cut and a distal femoral cut in 3° of valgus off the anatomical axis to keep the overall alignment in slight varus [3].

To balance the soft tissues in valgus osteoarthritis, multiple surgical techniques have been described: for knees that are tight in flexion and extension, Whiteside recommends releasing the LCL [51]. The popliteus tendon should be released for tightness in flexion [51]. For tightness in extension, Whiteside recommends a release of the ITB and the lateral posterior capsule [51]. For knees which are only tight laterally in extension, Whiteside recommends a release of the ITB and the lateral posterior capsule [51]. For tightness only in flexion, a release of the popliteus tendon followed by a release of the LCL is preferred [51]. Insall and Easley recommend releasing the ITB of Gerdy’s tubercle [53]. **Krackow** et al. describe a release of the ITB and the LCL first; a release of the popliteus tendon and the posterolateral capsule can be added if required [54]. Ranawat et al. recommend an “inside-out” technique using electrocautery at the level of the tibial cut surface from the posterior cruciate ligament to the posterior border of the ITB while preserving the popliteus if possible [3]. The ITB is lengthened with a “pie-crusting” technique as necessary from the inside [3]. Miyasaka et al. also recommend a “pie-crusting” technique to lengthen the ITB [55].

**Boettner** et al. report a cookbook standardized soft tissue release for valgus knees (release of the ITB and posterolateral corner) [7]. This approach produces the same excellent outcomes as surgical techniques, which are dependent on the surgeons’ judgment in terms of determining the extent of soft tissue release [7]. The authors recommend mechanical alignment targets for TKA and standardized soft tissue release, as published by the senior author [7].

## 2. Materials and Methods

We used the growing body of literature concerning alignment strategies for TKA in end-stage valgus osteoarthritis and our previous research articles on MA TKA with standardized soft tissue release for valgus knees to write this article. We performed a literature review using databases like PubMed and Google Scholar. We used search terms including “valgus osteoarthritis”, “kinematic alignment”, “inverse kinematic alignment”, “total knee arthroplasty” and “robotic knee replacement” to find relevant publications. The primary objective of this review paper is to report and summarize our current data and the current literature on the surgical techniques of TKA in patients with valgus osteoarthritis. This review will also outline targets for future research in this field.

## 3. Recommended Surgical Technique

Spinal or spinal–epidural anesthesia including intravenous sedation is utilized [7]. From incision until the cementation of the components, a tourniquet is used [7]. While the use of the tourniquet is not absolutely necessary, in valgus knees, the tourniquet improves the visualization of lateral soft tissues during inside-out soft tissue release.

(1) The preferred approach is the medial parapatellar approach [7]. While some authors have favored a lateral approach, the authors prefer a medial approach to avoid having wound closure over the area of lateral capsular release. The latter is used to improved patella tracking and or to release lateral soft tissues. Its close proximity to the lateral approach could increase the risk of wound drainage. The distal femoral cut is made in 5° using an intramedullary guide [7]. Cutting the femur at five degrees instead of three makes it easier to balance the knee by opening up that lateral joint space in extension. However, if one is not able to perfectly balance the knee (non-valgus), alignment needs to be either assured using technology or one should err on the side of slight varus alignment. The tibial cut is made at 90° using an extramedullary jig [7]. The posterior tibial slope is set according to the manufacturer’s recommendation [7]. In cases of evidence of medial joint space opening (Ranawat type II and III), attention is paid to minimize tibial resection [7]. Femoral component rotation is determined according to the transepicondylar axis and Whiteside’s line [7]. The tibia component is either aligned with the medial third of the tibial tuberosity or aligned with the anterior tibial spine. It is our belief that controlling the external rotation of the femoral component is very important in valgus knees, especially in patients with patella subluxation [7];
(2) A spacer block including alignment rods is utilized to validate the appropriate alignment of the distal femur and tibia cut before soft tissue release [7];
(3) Soft tissue release: With the knee in extension, using lamina spreaders to tension the extension space, the posterolateral capsule is released between the popliteus and the ITB [7]. Usually, a pop can be felt when the posterolateral corner is adequately released [7]. The LCL (in its midsubstance) and anterior lateral ligament (ALL) are included in this release [7]. A horizontal cut or pie-crusting through the ITB utilizing electrocautery at the level of the joint line is added until the extension space is balanced [7]. The popliteus tendon is maintained as a dynamic stabilizer [7];
(4) A spacer block is used for the evaluation of the extension and flexion gap [7]. If there is a remaining medial laxity and a constrained insert is needed, alignment is adjusted: through an additional 1–2 mm resection of the distal medial femoral condyle, alignment is adjusted into slight varus to ensure that there is no remaining valgus deformity [7].

Afterwards, a patella replacement is performed in the usual fashion with a cemented all-polyethylene patellar button [7].

**Figure 1:**
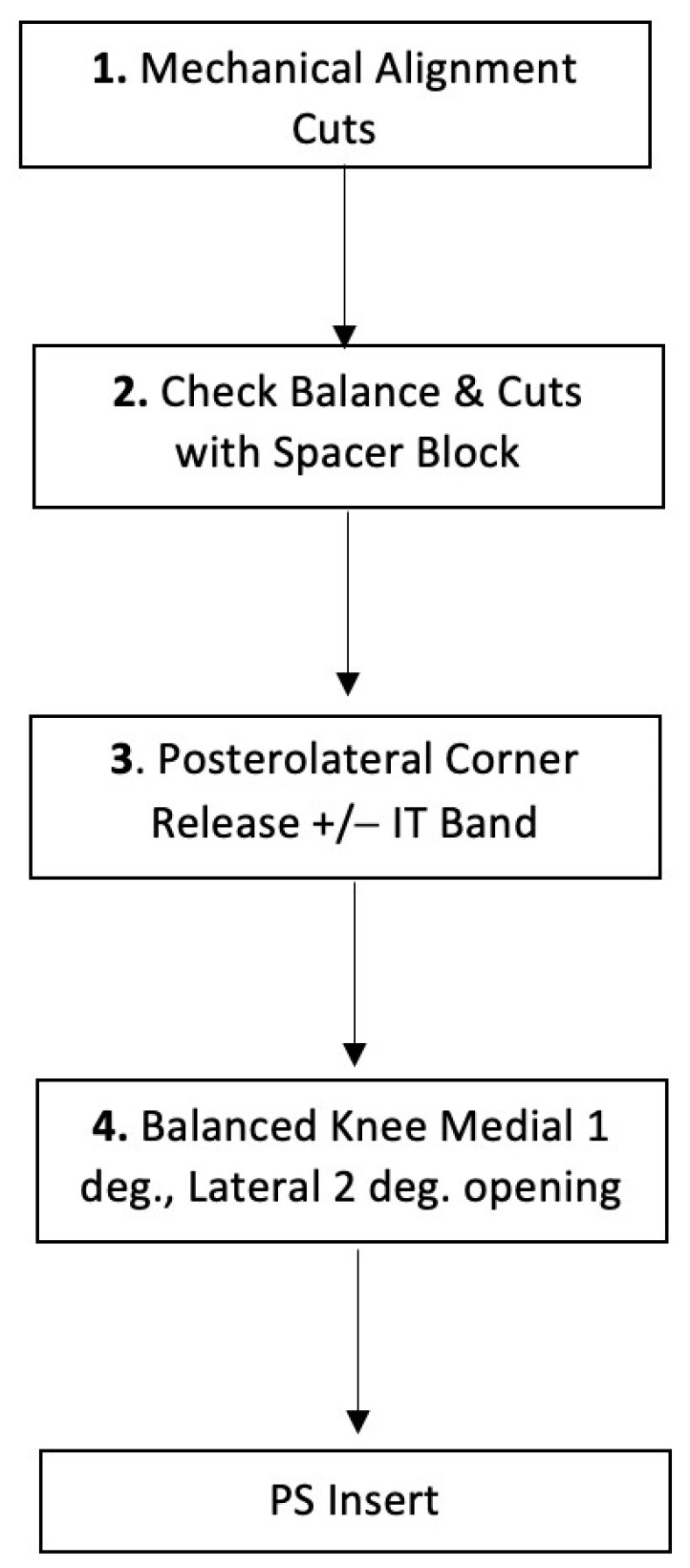
Our surgical approach to MA TKA in valgus knee.

In Figure 1, we summarize the recommended approach to the valgus knee.

## 4. Results

A total of 222 TKAs were carried out by the senior author (2008–2013) using a mechanical alignment target and standardized soft tissue release, as described above, in patients with valgus osteoarthritis of the knee [7]. Thirty-nine patients (17.6%) were not available for follow-up and two patients were excluded because they underwent TKA for posttraumatic osteoarthritis with valgus alignment [7]. Out of 181 TKAs, 144 (79.6%) received posterior stabilized tibial inserts and 37 (20.4%) constrained inserts [7]. The study reported excellent clinical outcomes after a follow-up of at least 2 years (range 24–87 months) for all patients with preoperative valgus alignment up to 25° [7]:

There was an increase in range of motion (ROM) from 4.7° preoperative flexion contracture (range 0–40) and 110° flexion (range 35–135) to 0.1° postoperative mean flexion contracture (range −5 to 10) and 128° flexion (range 100–140) [7]. Furthermore, the mean hip–knee–ankle alignment improved: the preoperative mean hip–knee–ankle alignment was 8.4° mechanical valgus (range 5.3–25.4) and the postoperative mean hip–knee–ankle alignment was 0.02° varus (range −2.9 to 4.1) [7]. The tibia component angle (MPTA) measured at 90.4° (range 86.1–93.7) [7]. The paper reported a significant improvement in WOMAC scores, VF-12, UCLA and VAS (*p* < 0.05) [7]. Revision surgery was performed on two patients (1.1%) [7]. There was no difference in functional outcomes between constrained versus posterior-stabilized TKAs (WOMAC, UCLA, VAS, VF-12, ROM) [7].

In a further study, 158 TKAs with the same mechanical alignment target and the same standardized soft tissue release for primary valgus osteoarthritis were analyzed in 135 patients [22]. Exclusion criteria were joint replacement on the same side and extraarticular deformity secondary to trauma [22].

A total of 124 knees (78.5%) were categorized as mechanically aligned [22]: HKA was 180° ± 3°. Valgus alignment was observed in 7.0% (11 knees) [22]. Within 180° ± 1.5°, 74 knees (46.8%) had a mechanical alignment and valgus alignment was seen in 39 knees (24.7%) [22]. Seventy percent were classified as group III (valgus aHKA, distal apex of JLO) [22] based on the coronal plane alignment of the knee (CPAK) classification [56].

MA TKA within the limit of 180°± 3° or 180° ± 1.5° showed no differences in outcome parameters (WOMAC, pain, UCLA, SF-12 and ROM) for the different preoperative CPAK phenotypes [22]. Furthermore, we were able to show that there were more beneficial values for postoperative UCLA (*p* = 0.011) and total SF-12 (*p* = 0.028) for MA TKA (boundary ± 1.5°) compared to TKA with remaining valgus alignment after surgery [22]. There were no differences in pain, mental and physical SF-12 components and ROM [22]. Furthermore, MA TKA (boundary ± 1.5°) showed better postoperative UCLA within CPAK group III compared to TKA with valgus alignment [22]. There were no reported differences in alternative parameters or among the remaining CPAK groups [22].

## 5. Discussion

The results of previous studies showed that mechanical alignment with standardized soft tissue release produces reproducible results for the valgus knee and that the preoperative phenotype does not influence the results of patients with valgus osteoarthritis operated with mechanical alignment targets [7,22].

Furthermore, we were able to show that in the largest CPAK group (group III (valgus aHKA, distal apex of JLO [56])), MA TKA produces more beneficial results compared to patients that are left in valgus alignment [22]. These data suggest that there is no functional advantage to preserving valgus alignment in patients with valgus osteoarthritis [22].

In contrast to findings in varus osteoarthritis of the knee [57,58], the results of the discussed studies propose that kinematic alignment (KA) with the aim of restoring the patient’s native alignment has no benefits and that MA TKA with standardized soft tissue release to balance the knee is the preferred treatment.

The senior author avoids KA (valgus alignment beyond 2 degrees of valgus) in valgus osteoarthritis because in his opinion, especially in patients with larger deformities and considerable medial opening or medial instability, KA increases the risk of secondary instability [22]. Trying to avoid soft tissue releases by using KA targets in patients with a stretched-out MCL will require more significant lateral condyle and tibia plateau bone resections compared to achieving balance using soft tissue release [22]. This can produce more significant valgus alignment compared to the native alignment [22]. More valgus alignment will in turn stress the already compromised MCL and might result in secondary instability [22].

Another important factor is that patients with valgus deformities of more than five degrees prefer to have their leg straightened, in contrast to patients with varus deformity, because “knocked knees” are less desirable cosmetically [22].

Furthermore, a retrospective study with 200 patients who received robotic-assisted TKA using functional alignment was able to show that kinematic alignment led to pronounced valgus and internal rotation of the femoral component in more than half of patients with valgus knees, after identifying intraoperatively recorded bone cuts and resulting joint line obliquities and comparing them to values obtained with a robotic computer simulation of kinematic alignment [59]. The authors of the study point out that creating a combination of excessive valgus and internal rotation on the femoral component increases the risk of patellofemoral maltracking [59]. This is a common reason for dissatisfaction after TKA [60,61]. In addition, increasing internal rotation to the femoral component can lead to changes in tibiofemoral kinematics and higher quadriceps requirements [62,63].

A finite element analysis found an increased failure rate when using kinematic alignment in valgus knees [64].

Kinematic alignment can be an option for patients with valgus deformity without medial opening with partially correctable deformities of less than 10 degrees [22]. In these cases, a decrease in the overall valgus alignment into a more acceptable range (less than 5 degrees) can be possible as a result of partial correctability [22]. In addition, there is less risk of secondary instability for patients with mild valgus deformity because there is no preoperative stretching and weakening of the MCL [22]. Readers have to take into consideration that any valgus stress during axial loading puts the knee at risk for injury to the MCL [65].

Long-term outcomes of KA TKA in valgus osteoarthritis are missing [66]. Comparing MA and KA TKA using patient-specific implants, one meta-analysis showed no differences between patients with native varus, valgus, or neutral alignment and no differences for early WOMAC and KSS combined scores [67].

On the other hand, many studies were able to show that MA TKA with standardized soft tissue release produces good clinical outcomes [3,7,52,68,69]: after a follow-up time of 11 years, Boyer et al. found only one revision for septic loosening and good clinical results for MA TKA [68]. Ranawat et al. was able to show that there was no late-onset instability in 490 knees after MA TKA for severe valgus osteoarthritis [3]. As described above, Ranawat proposed an overcorrection to varus alignment for valgus osteoarthritis [3]. One main advantage of this technique is that it avoids the undercorrection of the underlying deformity. With this technique, in more excessive valgus deformities, one can prevent a recurrence of the valgus deformity in patients with a relatively stretched out MCL. On the other hand, this overcorrection also complicates the surgery itself. It increases the need for soft tissue releases since ligamentous disbalance is increased when the knee is aligned in varus. In addition, patients with valgus alignment do not like significant varus alignment after surgery. Overcorrection also increases stress on the peroneal nerve. However, when leaving the joint relatively lax and using a hinge or constraint insert, the stress on the peroneal nerve can be minimized despite varus alignment. In general, excessive overcorrection of more than 3° of varus should be avoided since in the author’s experience, it will not result in a happy patient and increase load on the often osteoporotic medial tibia and femur.

New alignment strategies like FA and iKA, which were developed under the influence of new technologies like robotic surgery, create new opportunities for component alignment in valgus knees.

The principles of FA in valgus knees are, after customized preoperative planning, the reconstruction of the native coronal alignment without remaining varus or valgus alignment of more than three degrees and the reconstruction of dynamic sagittal alignment with boundaries of five degrees of neutral [45] followed by matching the anatomy with the size of the components and attaining the defined soft tissue laxity in extension and flexion by manipulating the components within the limits that have been established [45]. It has been shown for varus knees that, in contrast to KA, FA is more dependable in terms of preserving bone, the natural trochlea groove and tibiofemoral balance [70,71].

The following anatomic features of the valgus knee have been described: It has been shown that there is a hypoplastic lateral femoral condyle, resulting in a reduced mechanical lateral distal femur angle (mLDFA) of 85° ± 2.3° [72]. On the other hand, the mean medial proximal tibia angle (MPTA) is 90° ± 1.5° [73]. Springer et al. were also able to show a significantly reduced mLDFA in valgus OA with mean values of 84.6° for 50 female knees and of 85.4° for 50 male knees [74]. MPTA was significantly increased in these patients with valgus osteoarthritis (mean MPTA: 50 female knees: 89.7°, 50 male knees: 89.4°) [74]. While KA starts with the femoral side, the reduced mechanical lateral distal femur angle in valgus knees leads to oblique valgus cuts on the femoral side and could result in severe femoral valgus [48]. Consequently, with a KA approach, oblique varus cuts on a neutral tibia may be necessary for joint space balancing and will result in loss of bone on the medial tibia [48]. In contrast, iKA starts with resurfacing the tibial side [48]. Since the medial proximal tibia angle is approximately 90° in valgus knees, iKA will result in mechanically aligned tibial cuts and in similar bone resection on the medial and lateral side of the tibia to avoid overresection [48]. iKA in valgus knees will likely result in MA when it is combined with soft tissue releases and can be a promising approach for TKA in valgus knees. Furthermore, one important advantage of iKA compared to KA is that the femoral component can be implanted in external rotation [48]. This may improve outcomes of TKA in valgus osteoarthritis [48], as already described above.

This current review has the general limitations of a narrative review article concerning objectivity, the completeness of the literature search, and the interpretation of the findings [75]. Further limitations result from the limitations of the previous studies cited in the Results section.

## 6. Conclusions

In summary, the current literature supports MA TKA with soft tissue release as the gold standard for TKA in end-stage valgus osteoarthritis. MA TKA reduces the stress on an often already compromised MCL and reduces the risk of secondary instability. While using more sophisticated enabling technologies like robotic surgery might allow for aiming for very slight (1–2°) valgus alignment on the femoral side, in general, the risk of leaving the knee in valgus favors neutral mechanical alignment targets.

## 7. Future Directions and Clinical Implications

The increasing implementation of highly accurate alignment tools, including robotic surgery, will allow us to investigate different alignment strategies that might control and minimize the amount of valgus alignment. We believe that restricted inverse kinematic alignment that reproduces neutral mechanical alignment on the tibial side and adds soft tissue release prior to balancing the joint space with the femoral cut implementing functional alignment techniques holds potential for the future. Especially for more severe valgus knees, it is unlikely that the current recommendations will change over time; however, for mild valgus deformities up to 10 degrees of valgus, it is worthwhile to continue to investigate the benefits of a restricted inverse kinematic alignment strategies when using robotic surgery in combination with individualized soft tissue releases.

## Data Availability

Not applicable.
